# SAR ship target detection method based on CNN structure with wavelet and attention mechanism

**DOI:** 10.1371/journal.pone.0265599

**Published:** 2022-06-03

**Authors:** Shiqi Huang, Xuewen Pu, Xinke Zhan, Yucheng Zhang, Ziqi Dong, Jianshe Huang

**Affiliations:** 1 School of Information Technology & Engineering, Guangzhou College of Commerce, Guangzhou, China; 2 School of Information Engineering, Xijing University, Xi’an, China; Universiti Sains Malaysia, MALAYSIA

## Abstract

Ship target detection in synthetic aperture radar (SAR) images is an important application field. Due to the existence of sea clutter, especially the SAR imaging in huge wave area, SAR images contain a lot of complex noise, which brings great challenges to the effective detection of ship targets in SAR images. Although the deep semantic segmentation network has been widely used in the detection of ship targets in recent years, the global information of the image cannot be fully utilized. To solve this problem, a new convolutional neural network (CNN) method based on wavelet and attention mechanism was proposed in this paper, called the WA-CNN algorithm. The new method uses the U-Net structure to construct the network, which not only effectively reduces the depth of the network structure, but also significantly improves the complexity of the network. The basic network of WA-CNN algorithm consists of encoder and decoder. Dual tree complex wavelet transform (DTCWT) is introduced into the pooling layer of the encoder to smooth the speckle noise in SAR images, which is beneficial to preserve the contour structure and detail information of the target in the feature image. The attention mechanism theory is added into the decoder to obtain the global information of the ship target. Two public SAR image datasets were used to verify the proposed method, and good experimental results were obtained. This shows that the method proposed in this article is effective and feasible.

## 1. Introduction

Ship target detection of remote sensing images is very important for marine supervision, including illegal smuggling, traffic management, oil spill detection, piracy and fishery management [1–5]. The images used for ship detection mainly include reflected infrared, thermal infrared, optical and radar images. The radar imaging is different from other three methods, it can continuously obtain data at all time and all weather, and it has been widely used in many fields [6–9]. In recent years, with the launch of SAR satellites, such as Sentinel-1 [10], TerraSAR-X [11] and Gaofen-3, there are more and more high-resolution SAR images, which greatly improve the efficiency of ship detection in the process of ocean management [12–15].

The purpose of SAR image ship detection is to extract complete target area from complex background and to keep the better ship edge information. Appropriate ship segmentation algorithm can reduce the interference of complex background in SAR image. However, speckle noise caused by SAR coherence imaging mechanism is a common problem, which not only reduces image quality, but also affects image interpretation. Therefore, automatic and accurate ship segmentation on SAR images is still a challenging and important task.

There are many methods of ship target detection and segmentation based on SAR image. In general, these methods can be divided into two categories: supervised segmentation algorithm and unsupervised segmentation algorithm [16, 17].

Unsupervised algorithms do not require the prior knowledge like labels and ground truth. The unsupervised ship target detection algorithms mainly include threshold segmentation, clustering, active contour and Markov random field (MRF). The typical methods are Ostu method [18] and minimum error threshold method [19], which are effective for ships in black background images, but the segmentation effect is not ideal for SAR images with strong background clutter. Jin and Bai proposed the fuzzy c-means (FCM) clustering method in which the global distribution information of ship targets was fused in the form of Gaussian model [20]. Bai et al. designed a Markov random field target segmentation method combining symmetric information [21]. It is effective for pedestrian segmentation but it is opposite for ships with posture changes. Cao et al. used the watershed algorithm to segment and extract ship targets in the image [22]. Zhang et al. extracted ship targets from SAR images by combining ellipse constraint and gradient vector flow (GVF) snake model [23]. In target detection aspect, the unsupervised method is not superior to the supervised method in general, so the research and application of unsupervised method is not as good as the supervised method.

The supervised learning algorithm needs prior knowledge to train the model, and its purpose is to mine the hidden features in the data. At present, the popular supervised learning algorithm is the deep learning algorithms based on convolutional neural network structure. Deep learning algorithm can extract and select target features automatically, but traditional machine learning algorithm needs to create target features manually. Traditional supervised learning algorithms mainly include Bayesian algorithm, support vector machine (SVM) and K nearest neighbor (KNN). These methods are also widely used in the detection of ship targets [22, 24, 25].

With the improvement of computer hardware, deep learning has been more and more widely used in the field of computer vision and image processing. The general convolutional neural network (CNN) is to input the whole image into the network. After multi-layer network processing, finally the SoftMax layer obtains the category probability of the whole image. But the result is only to obtain the category of the image, and cannot achieve the semantic segmentation task of marking each pixel category. Fully convolutional networks (FCN) can overcome the weaknesses of CNN, and can achieve pixel-level image segmentation tasks [26–28] combined the masked region-based convolutional neural network (Mask R-CNN) and feature pyramid network (FPN) structure for the detection and segmentation of ship targets, and obtained good detection results [29]. applied the hole convolution to the CNN and increased the receptive field to save the features lost by pooling. Chen et al. proposed an end-to-end framework that combined deep convolutional neural networks and fully connected conditional random fields (CRF) [30, 31] designed a matching semantic segmentation network, which could detect the smallest bounding box of ship targets [32]. proposed a discriminator for judging the interference factors, such as sea fog, track and wave, improving the detection effect of ship targets [33]. used the DeepLabv3+ structure to effectively detect ship targets. It can be seen from the above documents that although the theories and methods based on deep learning can be used to achieve the detection of ship targets, these methods can be continuously optimized and improved. This is because these network structures are not only complex, but also have a large number of parameters. At the same time, for ship detection and segmentation of SAR image, they usually do not consider the interference and influence of SAR image speckle noise and other factors on ship detection effect. Of course, with the deepening of the network structure layer, especially for the deep structure network, the features in the intermediate feature graph become weaker and weaker. Therefore, through in-depth study of the existing ship target detection algorithms, we have found that reducing the network complexity, eliminating the influence of noise and making full use of the feature of the network middle layer can improve the network performance. Based on the above knowledge, this paper proposed a new CNN structure. It combines the theory of wavelet transform and attention mechanism, and realizes the effective detection of ship target in SAR image. Therefore, the new method is called WA-CNN ship target detection algorithm in this paper, namely WA-CNN method.

The new WA-CNN algorithm is an improvement based on the structure of U-Net network, so its basic structure is the same as that of U-Net network [34], which is composed of encoder and decoder. The WA-CNN algorithm considers the characteristics of SAR images and the advantages and disadvantages of U-Net network, so it not only can reduces the number of parameters and the layers of depth of U-Net network structure, but also can improves the efficiency of the network and the detection effect of ship targets in SAR image, which is suitable for SAR image processing. The main contributions and specific innovations or improvements of this paper include the following three aspects. (1) The quality of feature map can be effectively improved by adding wavelet transform. According to the characteristics of SAR image, the dual-tree complex wavelet transform is added in the coding stage of network structure to achieve the goal of removing speckle noise and improving the quality of feature map, so as to obtain better image maps. Speckle noise and directional sensitivity are the typical characteristics of SAR imaging, while the dual-tree complex wavelet transform has more directionality, which is suitable for SAR image processing and can extract more complete directional information. At the same time, through multi-scale decomposition, speckle noise is usually concentrated in high-frequency sub-images, and selecting low-frequency sub images can well filter out a lot of speckle noise. (2) Attention mechanism is introduced to improve the utilization of middle layer features and the extraction rate of global information. In the decomposition layer, some convolution networks not only extract local information, but also have low utilization of features. Therefore, the WA-CNN algorithm solves this problem by introducing attention mechanism to improve the effective utilization of features. The attention mechanism layer is created in the decoder, which improves the utilization of the middle layer, and makes full use of the global information in the feature calculation process to make the acquired information more accurate. (3) It designs wavelet decomposition pooling layer in the network structure, which not only greatly reduces the number of depth layers and parameters of U-Net network, but also improves the efficiency of the network.

The rest of this paper was organized as follows. Section 2 introduced the principle and implementation process of the WA-CNN algorithm. Section 3 explained the experimental data, experimental design and performance evaluation indicators. The experimental results were analyzed and discussed in detail in Section 4. And the summary of this paper and the prospect of future research work were introduced in Section 5.

## 2. Principle description of WA-CNN algorithm

### A. Construction of WA-CNN network structure

**Fig 1:**
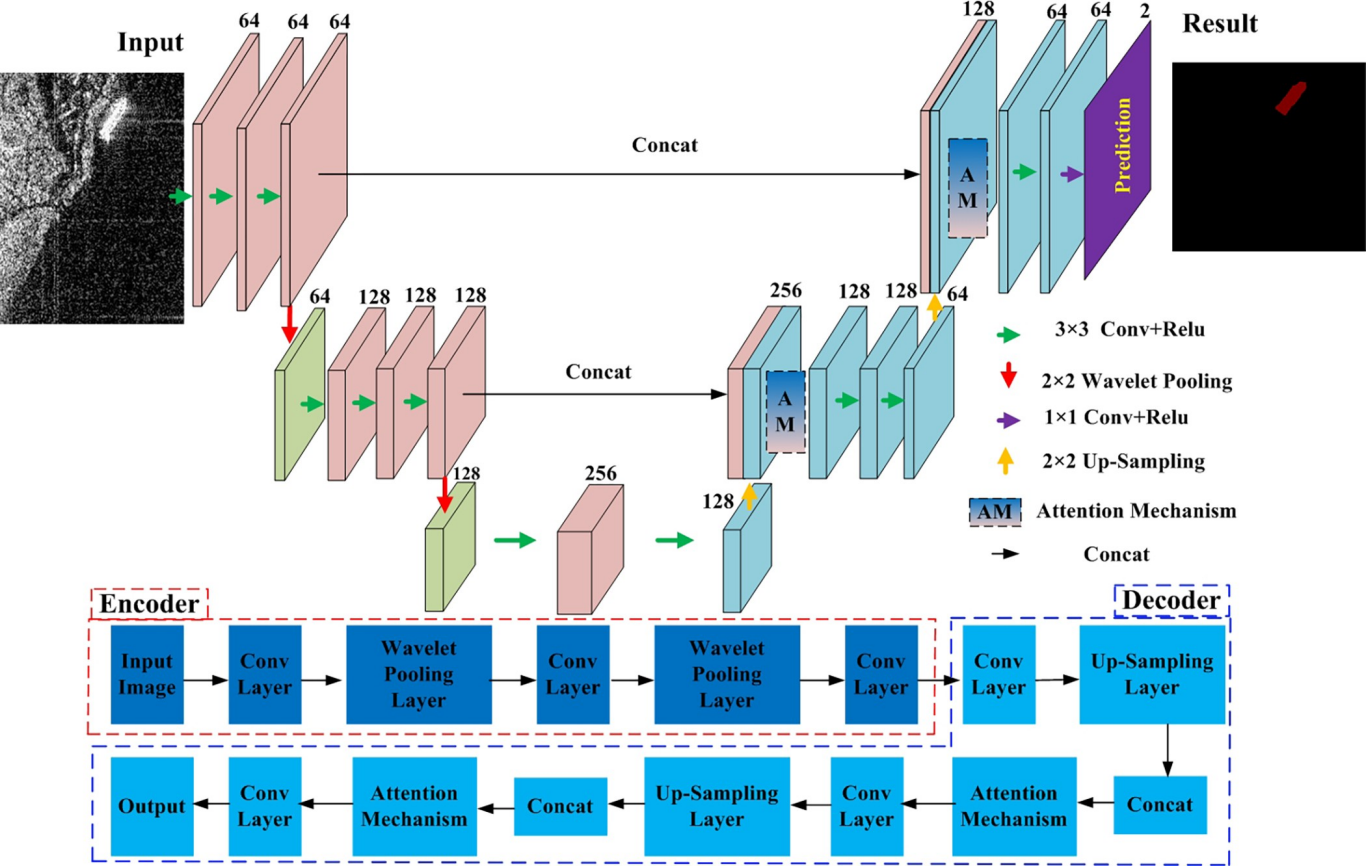
The overall framework of the WA-CNN network.

The structure of the WA-CNN network is shown in Fig 1. Its basic structure is similar to a U-Net network structure, with an encoder and decoder. The structure of the encoder part is composed of three stages, and each stage is connected by a pooling layer. Because the dual-tree complex wavelet transform (DTCWT) has good performance in direction selection, redundancy and reconstruction, the pooling layer in the encoding process uses DTCWT to perform the pooling operation, i.e. the wavelet pooling. Its purpose is to reduce the influence of speckle noise of SAR images, and to preserve more structural information, such as edges, endpoints and corners. The structure of the decoder part is also composed of three stages, each stage is connected by an up-sampling layer, and its function is to enlarge the output feature map to twice the input. An attention mechanism is constructed in the decoding process, which can better perform feature extraction and fusion, and which is beneficial to extracting global information.

To improve the computing performance of the network, the original input image is cropped into 256×256. In the first stage of the encoder, the convolution layer performs three convolution operations. The size of the convolution kernel is 3×3 and there are 64 convolution kernels. Therefore, the depth of the feature map is 64. In the second stage of the encoder, firstly, the input feature map is processed by wavelet pooling, and the wavelet pooling layer obtains the feature map whose size is half of the input feature map and depth is 64. Next, the result of the wavelet pooling layer is subjected to three convolution operations. The size of the convolution kernel is still 3×3 and there are 128 convolution kernels, so the convolution layer can obtain a feature map with a depth of 128. In the third stage of the encoder, through the processing of the wavelet pooling layer, a feature map with a size of half of the input feature map and a depth of 128 can be obtained. The size of the convolution kernel is still 3×3, and its number is 256, so a feature map with a depth of 256 can be obtained. The output feature map of the third stage of the encoder is used as the input image of the first stage of the decoder. The decoder is symmetrical to the encoder. However, the feature map obtained by the up sampling layer will be fused with the feature map of the encoder in order to increase the number of channels of the feature map, and an attention mechanism is also introduced during feature fusion. Through the processing and calculation of attention mechanism, the output feature map can contain the global information. In the last stage of the decoder, a feature map with a depth of 64 performs a convolution operation with two convolution kernels and their size is 1×1. In addition, the convolutional layer obtains a predicted feature map with two channels, which represents the ship target and background, respectively. The implementation of wavelet pooling and attention mechanism in the network structure will be introduced in detail in next sub-sections B and C.

### B. Principle of wavelet pooling

The convolutional layer in CNN extracts the most basic visual features, such as endpoints and corners, and these features form high-level abstract features in the subsequent layers. It is very important to preserve these features for segmentation effect. However, the inherent speckle noise in SAR image affects the feature extraction, further affects the detection and segmentation of ship targets. The simple max pooling processing in CNN network often loses some detail features. To reduce the influence of speckle noise and to save more detailed feature information, wavelet pooling layer is introduced to replace the traditional max pooling layer in CNN network. In the wavelet pooling layer, DTCWT is chosen for pooling operation, because it has the following advantages, such as approximate invariance, efficient sequential computation, limited redundancy, perfect reconstruction and good directional selectivity. The feature image generated by convolution layer can be transformed by DTCWT to generate two low-frequency coefficient sub-images *LL*_1_ and *LL*_2_, and six high-frequency coefficient sub-images in different directions *HL*_1_, *HL*_2_, *LH*_1_, *LH*_2_, *HH*_1_ and *HH*_2_, corresponding to the direction of ±15°, ±45° and ±75°, respectively. The average value of the sum of the two low-frequency coefficient sub-images is used as the output of the wavelet pooling layer.

**Fig 2:**
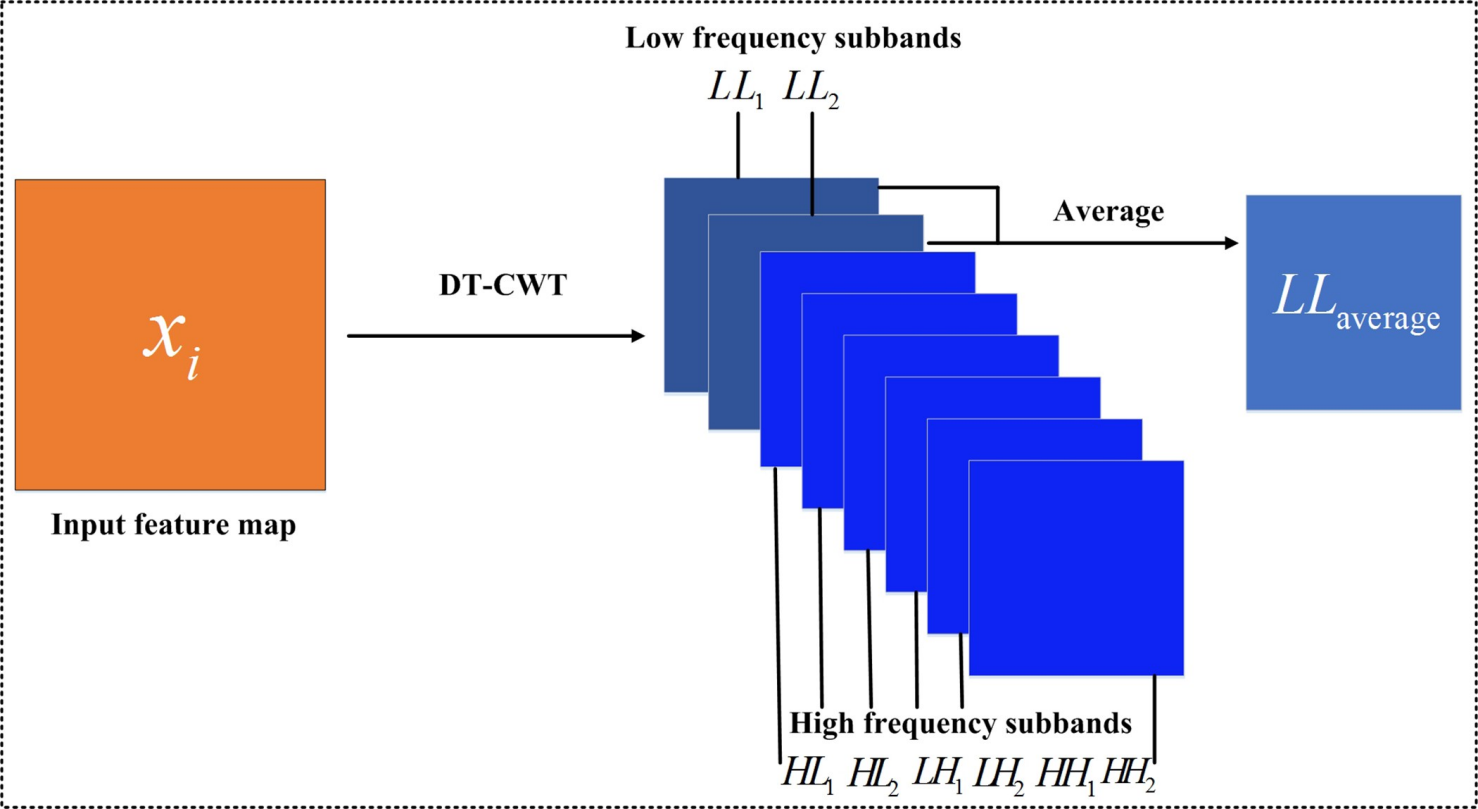
Schematic diagram of wavelet pooling layer.


By introducing DTCWT into the deep convolutional network structure, the structural feature information of the input layer is preserved by its low-frequency coefficient sub-images through specific rules, and the speckle noise in the SAR image is suppressed by its high-frequency coefficient sub-images. Similar to the max pooling layer, the input of the wavelet pooling layer is the output of the convolutional layer, as shown in Fig 2. In the wavelet pooling layer, after each input feature map is transformed by DTCWT theory, eight sub-image feature maps can be obtained, as shown in Eq (1).

$$f(x_{i})=\{LL_{1},LL_{2},LH_{1},LH_{2},HL_{1},HL_{2},HH_{1},HH_{2}\}$$ (1)

Where *x*_*i*_ denotes the input feature image. Then low-frequency sub-images *LL*_1_ and *LL*_2_ are averaged to obtain their average value *LL*_*average*_, which is used as the output of wavelet pooling layer. The definition is given as follows.


$$LL_{average}=\frac{LL_{1}+LL_{2}}{2}$$ (2)

**Fig 3:**
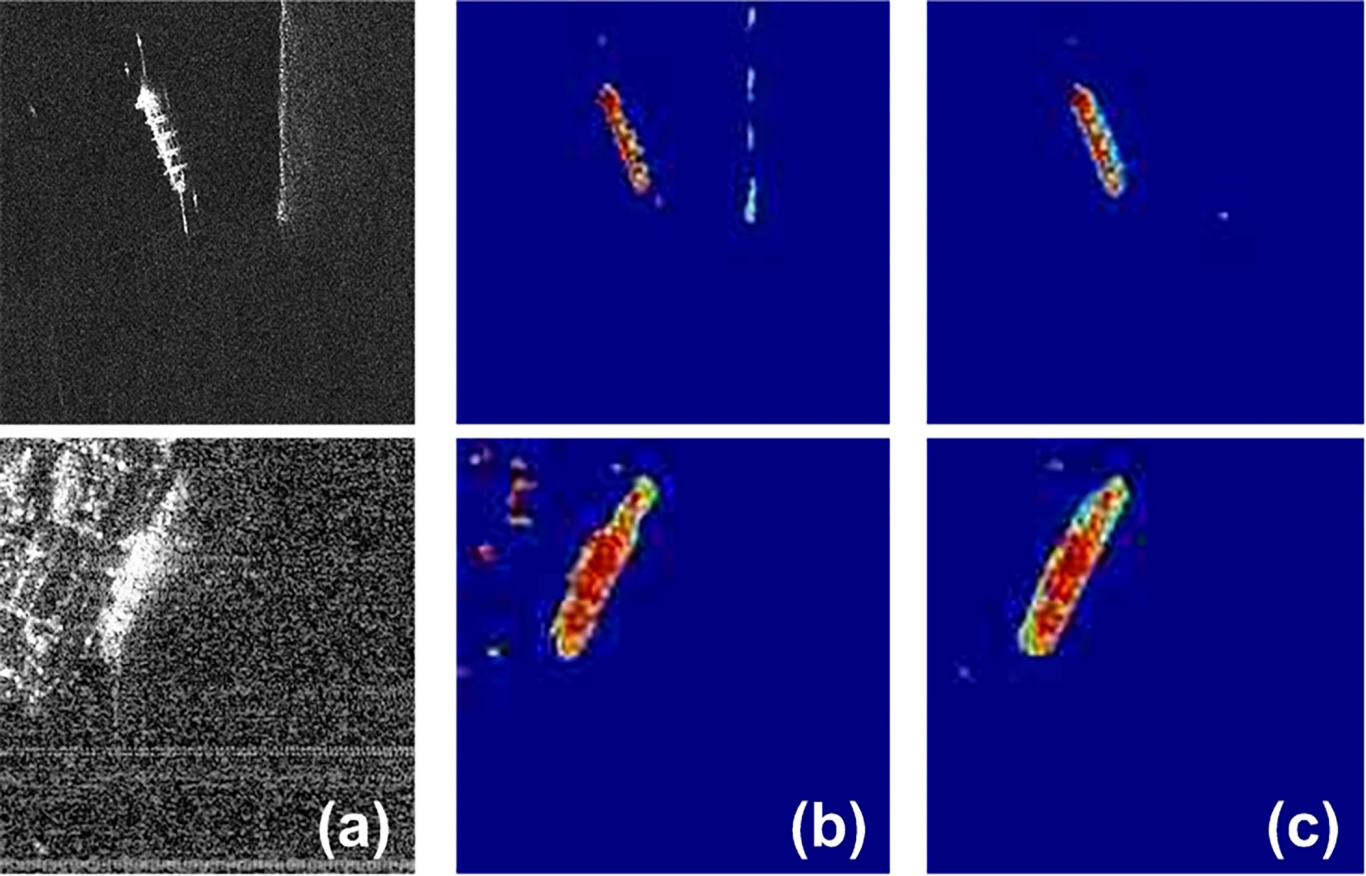
Feature maps produced by different pooling layers. (a. original SAR ship image, b. feature map processed by CNN max pooling layer, c. feature map processed by wavelet pooling layer in WA-CNN).

In fact, in the wavelet pooling layer shown in Fig 2, the low-frequency coefficient sub-images generated by DTCWT can reduce the influence of SAR image speckle noise, so as to maintain good structural features, and it is very beneficial for target segmentation in SAR image. Fig 3 shows the feature maps obtained by the max pooling layer in CNN and the wavelet pooling layer in WA-CNN. Fig 3(A) shows two different original SAR images, containing one ship target. Fig 3(B) is the feature map obtained by CNN, and the feature map shown in Fig 3(C) is obtained by WA-CNN. It can be seen in Fig 3 that the feature map extracted by WA-CNN can retain more feature information, the shape of the ship target is more complete, and the noise interference is reduced.

### C. Capturing features based on attention mechanism

In traditional CNN, deep level feature extraction is realized by convolution operation between the input image and convolution kernel. However, because the convolution kernel is relatively small, compared to the original image or the target area, part of the information cannot be effectively extracted, which will affect the final detection or segmentation effect of the target area. With the number of convolutional layers adding, this defect becomes more and more serious. For the detailed structure of the ship target in the SAR image, it is very important to solve this problem. This problem can be overcome by adding an attention mechanism to a suitable location in the network.

**Fig 4:**
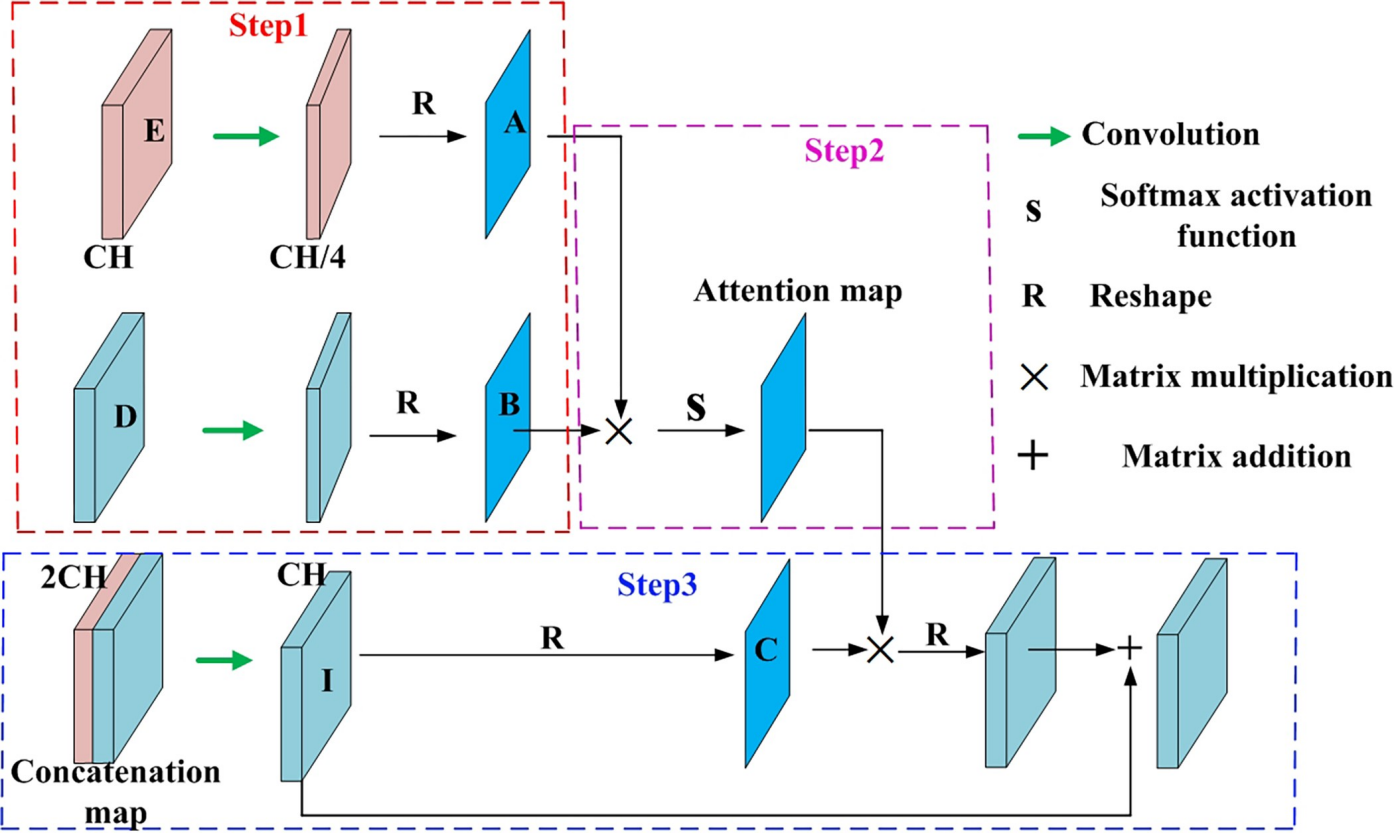
Schematic diagram of feature extraction by attention mechanism.

In the WA-CNN algorithm, the process of using the attention mechanism to improve the accuracy of ship target detection in SAR images mainly includes three steps, namely similar feature extraction, feature similarity calculation and original features enhancement. The specific principle block diagram is shown in Fig 4.

Step 1: Similar feature extraction. Firstly, the features in the original feature map are divided into three spaces A, B and C. The distribution of spatial features of A and B is similar to that of C space. Therefore, the features of C space can be enhanced by the features of A and B spaces. The features of the encoding part and the decoding part in the whole network generate A and B spaces respectively. The features of encoder and decoder are fused in series on the channel, and then the concatenation features are obtained and used to generate the features in C space. Three spaces are generated by convolution layer. In Fig 4, the feature maps in the encoding and decoding process are represented by E and D. The number of channels is reduced by convolution operation, and the channel is reduced to 1/4 of the original feature, $\{E,D\}\in {\mathbb{R}}^{W\times H\times CH}$. Here *W*, *H* and *CH* represent the width, height and channel number of the feature map, respectively. To calculate feature similarity, three-dimensional features need to be reduced to two-dimensional. Then the three-dimensional features with reduced channels are reshaped into two-dimensional feature matrices **A** and **B**.

Step 2: Feature similarity calculation. The similarity matrix between **A** and **B** is realized by dot product operation.


$$G(•)=A^{T}B$$ (3)

The similarity matrix is activated by the SoftMax function, and the attention feature map $F\in {\mathbb{R}}^{\left(W\times H)\times (W\times H\right)}$ is obtained.


$$F_{ij}=\frac{\exp\left(A_{i}\times B_{j}\right)}{\sum_{i=1}^{W\times H}\sum_{j=1}^{W\times H}\exp\left(A_{i}\times B_{j}\right)}$$ (4)

Where **F**_*ij*_ represents the correlation between the *i*th feature in matrix **A** and the *j*th feature in matrix **B**. The attention feature map is a coefficient matrix with a value between 0–1, which is used to enhance the C space and reflects the similarity between any two points in the matrix **A** and **B**.

Step 3: Original features enhancement. The encoding feature and the decoding feature are stacked on the channel to form a concatenated feature, and its dimension becomes *W*×*H*×2*CH*. Concatenated features reduce the number of channels through the convolutional layer to obtain a new feature map *I*, $I\in {\mathbb{R}}^{W\times H\times CH}$. To enhance the original feature, feature map *I* is reshaped into matrix **C**. The matrix **C** and the two-dimensional attention map are multiplied to obtain an enhanced feature map. The enhanced feature map is reshaped from two-dimensional to a three-dimensional feature map through the convolutional layer, and its dimension is ${\mathbb{R}}^{W\times H\times CH}$. It can be regarded as a coefficient matrix used to enhance the original features, and its mathematical model is as follows.


$$Q_{j}=\varepsilon \sum_{i=1}^{W\times H}\left(F_{ij}C_{j}\right)+I_{j}$$ (5)

Where *Q*_*j*_ is the *j*th feature generated by the attention mechanism, and *ε* is a coefficient used to measure the proportion of attention features in all features. Finally, the attention feature map and the original feature map are added to obtain the final enhanced feature map.

## 3. Experimental design and description

### A. Data introduction

The experimental data used in this experiment comes from two public datasets, namely, SSDD [35] and SAR-SHIP-SET [36]. There are 1160 images and 2356 ships in the SSDD dataset. The size of the images in the dataset is approximately 500×500. The data is mainly acquired by satellites such as RadarSat-2, TerraSAR-X and Sentinel-1, including four polarization modes of HH, HV, VV and VH, with a spatial resolution of 1m-15m. There are ship targets in large areas of sea and coastal areas. To improve the training speed of the network, the original image is cropped into a small image with the size of 256×256. For comparing the experimental results of different methods under the same conditions, the allocation of the training set and test set is not specified. Because the number of SSDD datasets is relatively large, half of the training data and half of the test data are allocated to verify the experiments.

The SAR-SHIP-SET dataset contains 210 scenes of SAR ship images. There are 102 scene images acquired by Chinese Gaofen-3 satellite and other 108 scenes images are from sentinel-1 satellite. To facilitate calculation and processing, the original SAR image is cropped into an image of size of 256×256, which contains 43819 ship slice images in total. The entire data set is randomly divided into the training set and test set, and their proportions are 70% and 30%, respectively.

### B. Training parameter setting

The WA-CNN network uses batch iterative methods to complete the network training. During the training process, the batch size is set to 20, and the number of iterations is 1000. The learning rate is set and updated according to the number of iterations. The number of iterations is less than 200 as the first stage, and the learning rate is set to 0.001. When the number of iterations is greater than or equal to 200 but less than 800, it is the second stage, and the learning rate is 0.0005. The third stage is when the number of iterations is greater than or equal to 800, and the learning rate is 0.0001.

### C. Evaluation index

For ship detection in SAR images, there are only two types of final classification results for each pixel, namely ship pixel and non-ship pixel, and they are also called the positive class and negative class. When the detection result is compared with the true value, there will be two kinds of correct classification and wrong classification, which are recorded as true and false, respectively. If a pixel of ship is detected as a ship, this pixel is the true positive class (TP); if it is detected as a non-ship class, then this pixel is called a false negative class (FN). If a non-ship pixel is correctly detected as a non-ship class, then this pixel is called a true negative class (TN); if it is incorrectly detected as a ship class, it is called a false positive class (FP). If the values of the true positive class and the true negative class are larger, the accuracy of the correct classification of the ship target is higher.

The evaluation indexes of target detection performance include sensitivity (SE), specificity (SP), accuracy (ACC) and area under curve (AUC) of the receiver operating characteristic curve (ROC). In addition to AUC, the mathematical definitions of other three evaluation parameters are as follows.


$$SE=\frac{TP}{TP+FN}$$ (6)

$$SP=\frac{TN}{TN+FP}$$ (7)

$$ACC=\frac{TP+TN}{TP+TN+FP+FN}$$ (8)

The parameter SE denotes the segmentation performance of ship target pixels, SP denotes the segmentation performance of non-ship target pixels, and ACC represents the segmentation performance of the entire image pixel. AUC is the area under the ROC curve and the maximum value of the area is one. The larger the value of AUC is, the better the segmentation effect of the network is. It should be noted that the evaluation indexes SE, SP and AUC are evaluated from the perspective of machine learning classification, that is, from the accuracy of deep learning model prediction, while ACC parameter is evaluated from the field of image pixel classification (image segmentation). Although they have different levels, the purpose of evaluation is the same.

## 4. Analysis and discussion of experimental results

To verify the feasibility and effectiveness of the WA-CNN algorithm, verification experiments were carried out with SAR images from SSDD and SAR-SHIP-SET databases, and comparative experiments were also done with other networks such as FCN, U-Net and DeepLabv3+. The evaluation indexes mentioned above are used to analyze the experimental results obtained by different methods, and the performance of each algorithm is discussed.

### A. Experiments with SSDD data set

**Fig 5:**
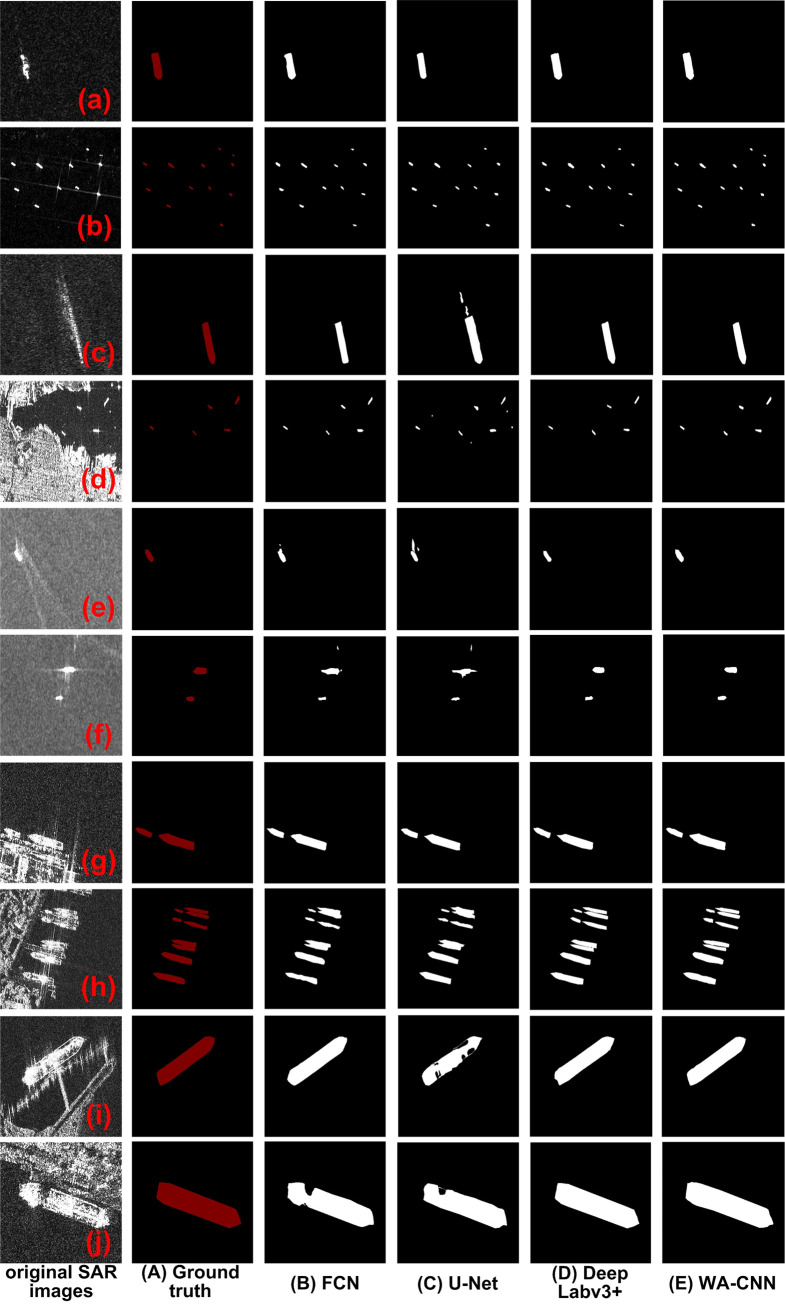
Ship detection results of different algorithms with the SSDD data set. (a-j represents the SAR images of different scenes, A is the ground truth, and B-E are the processing results of the FCN, U-Net, DeepLabv3+ and WA-CNN algorithms).

The ship target detection and segmentation experiments were carried out with the SAR images in the SSDD database. The experimental data and experimental results are shown in Fig 5. The original SAR images are shown in Fig 5(A)–5(J). These experimental images contain ship targets and different scenes. For example, the imaging area includes near shore and deep sea; there are not only SAR images with lots of speckle noise, but also SAR images with dark background; there are both high resolution and low resolution SAR images. Fig 5(E) and 5(F) contain a large amount of strong speckle noise, while other images have weak speckle noise. The wake of the ship target in Fig 5(C) and 5(E) is obvious. There are multiple ship targets in Fig 5(B)–5(D) and 5(H). Fig 5(A)–5(F) are SAR images with medium and low resolutions, and all the ships in the image are in the state of separation and have no contact with other objects around them. But Fig 5(D) is a SAR image near coast, and there are a lot of land scenes in the image. Fig 5(G)–5(J) are high-resolution SAR images, and the ship targets in these SAR images are docked in the harbor, so they are connected with the surrounding buildings. The ground truth of ship target in SAR image is shown in Fig 5(A), and the experimental results obtained by FCN, U-Net, DeepLabv3+ and WA-CNN are shown in Fig 5(B)–5(E), respectively.

It can be seen in Fig 5(B) that when the FCN method is used to detect Fig 5(A), the boundary of the ship is missing. In Fig 5(B), there are some omissions in ship target detection. In Fig 5(E) and (5F), there is a strong speckle noise which is mistakenly detected as a ship target. In Fig 5(H), the boundaries of the detection results of adjacent ships are not obvious. In Fig 5(J), the detected ship target is missing and incomplete.

The result of U-Net method is shown in Fig 5(C). The experimental results obtained by U-Net are very similar to those obtained by the FCN method, which is not ideal. For example, in Fig 5(A), the detection effect is not bad, and the boundary detection of the ship is relatively complete. There is a missed detection phenomenon in Fig 5(B), the waves generated by the ship’s traveling are mistakenly detected as ships in Fig 5(C), and some islands are detected as ship targets in Fig 5(D). For the SAR images shown in Fig 5(E) and 5(F), the speckle noise is relatively strong, resulting in false detection, and the noise is mistakenly detected as a ship. In Fig 5(H), the boundaries of adjacent ships are also not distinguished. For the high-resolution SAR images shown in Fig 5(I) and 5(J), the detection results are incomplete and missing.

The image shown in Fig 5(D) is the detection result of DeepLabv3+. In general, the edges of ships are relatively complete, and they are less affected by speckle noise. The detected ship targets are well distinguished from ports, coasts and islands, and there is no missing phenomenon in the detection of ships under high resolution. The only disadvantage is that the small ship target with weak scattering in Fig 5(B) is missed.

From the visual effect, the experimental results obtained by the WA-CNN algorithm are very good, which is shown in Fig 5(E). It can not only recognize ship targets in SAR images of different scenes accurately, but also have complete edge structure. Whether it is a low resolution or high resolution SAR image, the ships can be effectively detected, without missing detection or false detection, and it can overcome the influence of speckle noise, wave, port, island and adjacent ships.

**Fig 6:**
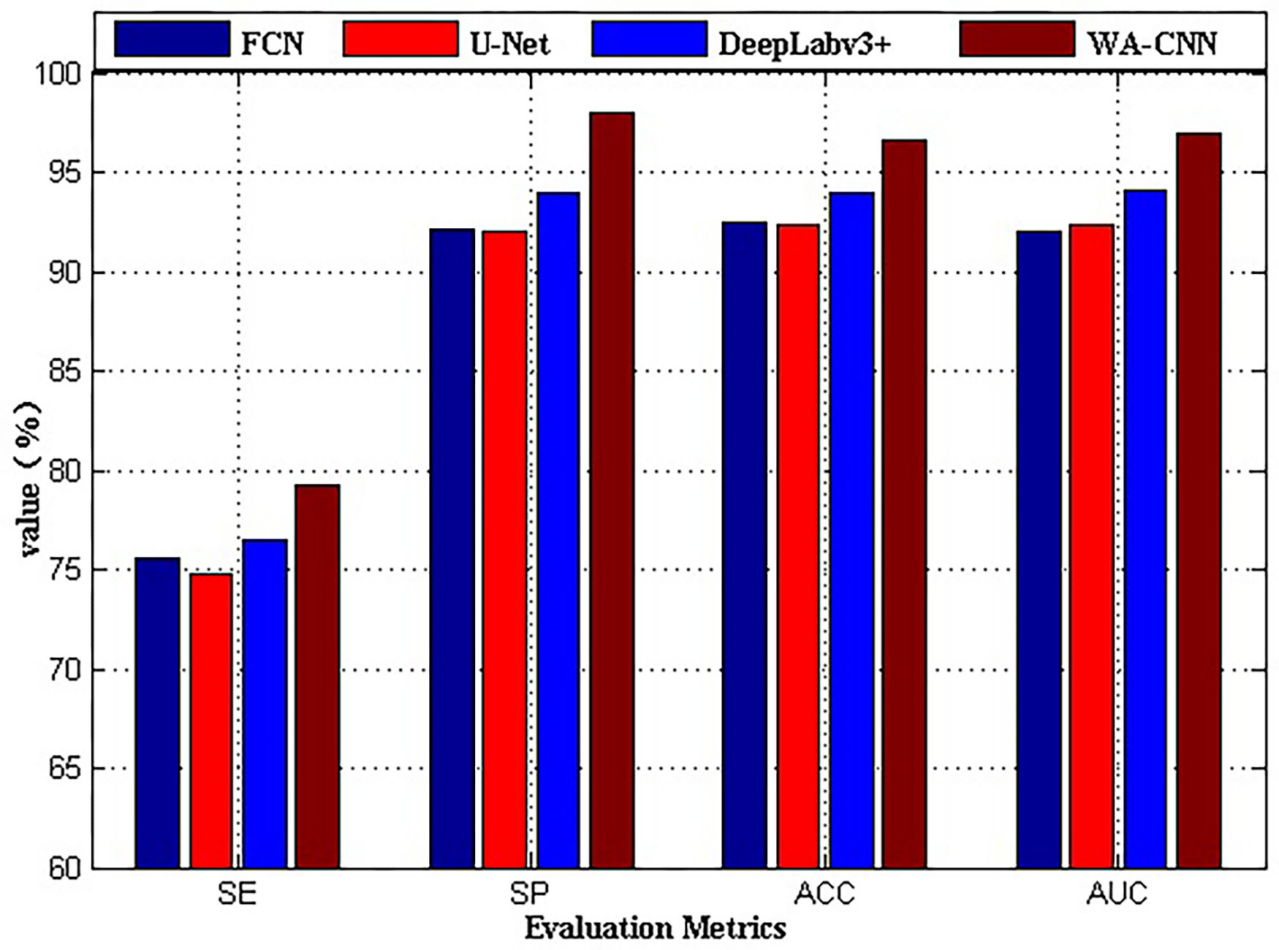
Comparison of performance indicators of different algorithms on SSDD data set.

The analysis of the above experimental results shows that for the SAR image ship detection of using the SSDD data set, the best detection effect is WA-CNN algorithm, next DeepLabv3+, finally FCN and U-Net. To further compare and analyze the performance of these four methods, four evaluation parameters of SE, SP, ACC and AUC are selected for specific description, and the experimental results are shown in Fig 6. Here, the abscissa represents different evaluation parameter indexes, and the ordinate is the value of each index. It is worth noting that the parameter values of each index in Fig 6 are not obtained from a single image, but it is the mathematical average of the same evaluation parameter values of all images processed by the same method. For example, the second value of the parameter SE (from left to right) in Fig 6, it is obtained from all images processed by the U-Net algorithm. Firstly, the SE value of each image shown in Fig 5(C) is calculated, then all values of parameter SE perform summation and mean operations, and the result is the final value of the parameter SE. The acquisition process of other parameter values is similar. The parameter values shown in Fig 6 are calculated using the result images obtained by different algorithms in Fig 5.

As can be seen in Fig 6, for the four evaluation indexes SE, SP, ACC and AUC, the calculated value of the result image obtained by WA-CNN algorithm is significantly higher than that of other three methods. This shows that WA-CNN algorithm can effectively and completely detect ship targets in SAR images with SSDD dataset, and the effect of parameter evaluation is also the best. Then the algorithm with better performance is DeepLabv3+, and finally FCN and U-Net. It is known in Fig 6 that the performance indicators of the FCN algorithm and the U-Net algorithm are very close, indicating that their ship detection effects are very similar, as shown in Fig 5(B) and 5(C).

### B. Experiments with the SAR-SHIP-SET data set

**Fig 7:**
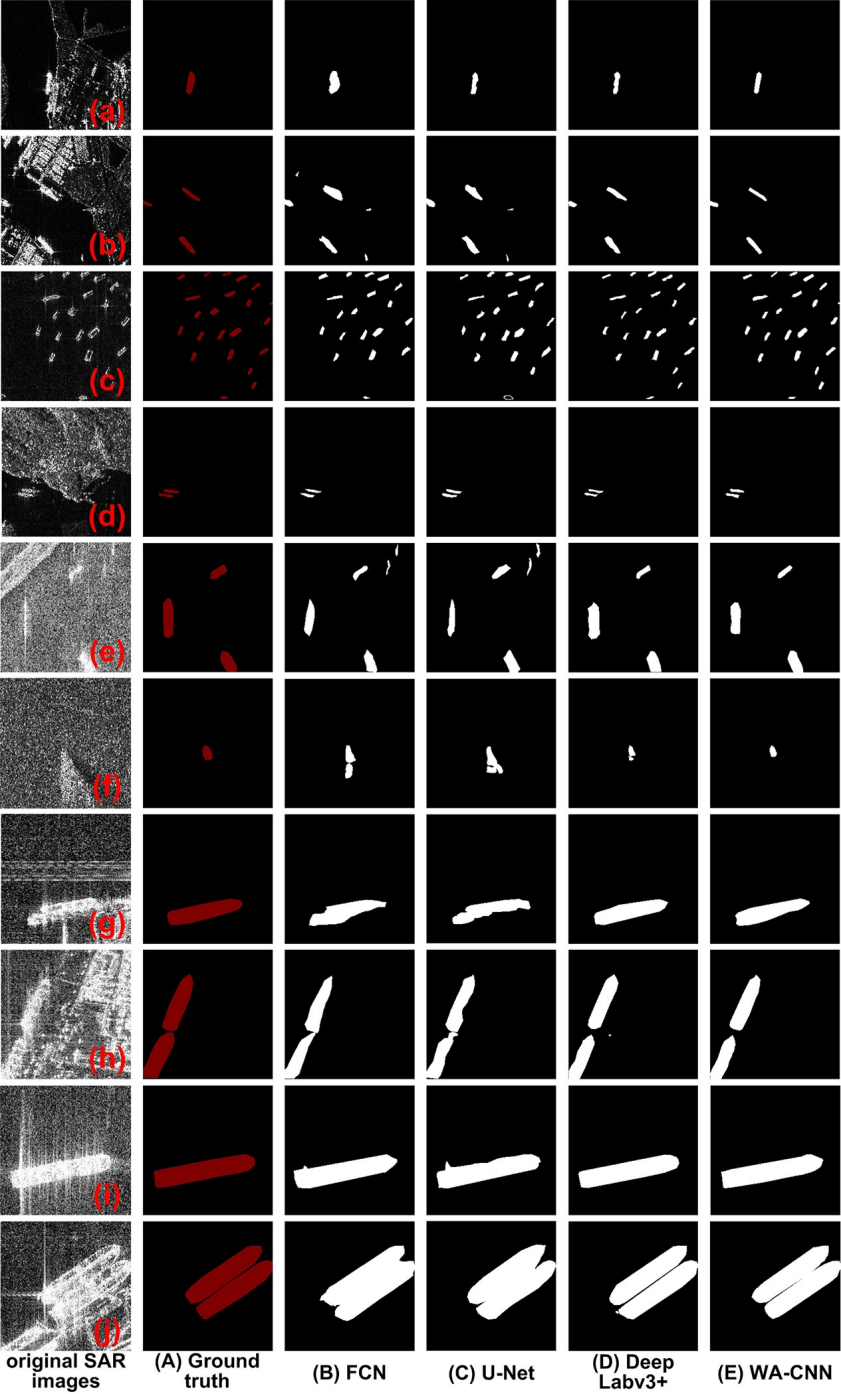
Ship detection results of different algorithms on the SAR-SHIP-SET dataset. (a-j represents the SAR images of different scenes, A is ground true, B-E are the results obtained by the FCN, U-Net, DeepLabv3+ and WA-CNN algorithms, respectively.).

To verify the processing effect of the WA-CNN algorithm on other SAR image data, the data of SAR images in the SAR-SHIP-SET data set is used to carry out relevant experiments. Because the types of ship targets in the SAR images collected by this dataset are more abundant, and the imaging scene is more complex. The experimental results and experimental images were shown in Fig 7. Fig 7(A)–7(J) show the original SAR images of different scenes. Those SAR images shown in Fig 7(A) and 7(B) are from the Sentinel-1 satellite, and other SAR images are acquired by the Gaofen-3 satellite. Fig 7(C)–7(F) belong to medium resolution images, and the imaging areas are near coast and large waves. Fig 7(G)–7(J) are high resolution SAR images, and there are many interference factors. Fig 7(A) shows ground truth, and Fig 7(B)–7(E) show experimental results obtained by FCN, U-Net, DeepLabv3+ and WA-CNN algorithms, respectively.

In Fig 7, the detection result of the FCN algorithm is shown in Fig 7(B). For Fig 7(A), the FCN algorithm mistakenly detects some ports as ships, and in Fig 7(B), it detects islands as ship targets. In Fig 7(C), two small ship targets are missed. For Fig 7(E) and 7(F), strong noise and waves are detected inside the ship. In Fig 7(H) and 7(J), the edge extraction of ships is not ideal. Not only the edges are missed, but also the edges of adjacent ships are very difficult to distinguish. It can be seen that the FCN algorithm has a poor detection effect in the SAR image ship target with SAR-SHIP-SET dataset. The images shown in Fig 7(C) are obtained by U-Net algorithm. Obviously, the detection effect of U-Net algorithm and FCN algorithm is very similar. It not only mistakenly detects targets such as ports, islands, noise and waves as ships, but also often fails to detect small ships or the edges of detection targets are not clear. Therefore, for U-Net and FCN algorithms, their detection effect is relatively poor in SAR images. Fig 7(D) shows the detection result of DeepLabv3+ algorithm. It can be seen that the effect of DeepLabv3+ is generally better than that of U-Net and FCN, but for the processing of Fig 7(F)–7(H), there is an error classification. For Fig 7(A)–7(E) and 7(J), the edge and shape of the extracted ship have a large deviation. The experimental results of WA-CNN algorithm are shown in Fig 7(E). It can be seen that WA-CNN algorithm can detect all kinds of ships in Fig 7(A)–7(J), the effect is also good and is very close to the ground truth.

**Fig 8:**
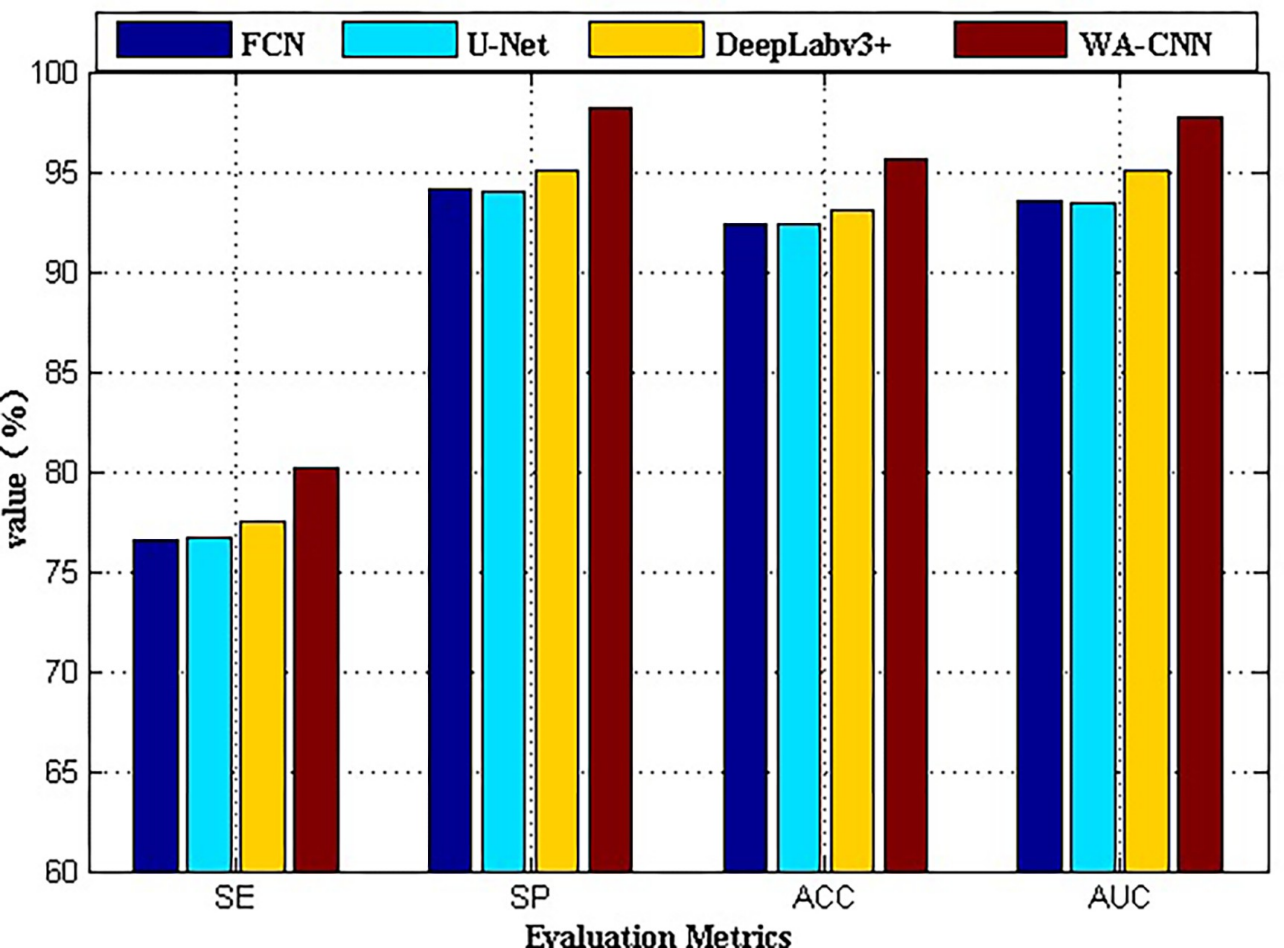
Performance comparison of different algorithms on SAR-SHIP-SET data set.

The above content mainly discusses the detection effect of different algorithms from subjective vision. Next, some parameter evaluation indexes such as SE, SP, ACC and AUC will be utilized to evaluate the performance of different algorithms on SAR-SHIP-SET data. The results are shown in Fig 8, and the coordinate meaning of it is the same as that of Fig 6. The abscissa denotes the evaluation parameters (i.e. SE, SP, ACC and AUC), and the ordinate represents their values. Each parameter contains four algorithms used for experiments, from left to right are FCN, U-Net, DeepLabv3+ and WA-CNN. The process of obtaining parameter value in Fig 8 is exactly same as that of Fig 6, and its physical meaning is also same. The value in Fig 8 is calculated by the experimental result image shown in Fig 7. It can be seen in Fig 8 that regardless of the evaluation index value, the result of WA-CNN algorithm is higher than that of other, and the other three methods have little difference in value. The parameter values obtained by the DeepLabv3+ algorithm are slightly larger than those obtained by FCN and U-Net algorithms. At the same time, different performance indicators of FCN and U-Net are relatively close. The law reflected in Fig 8 is the same as that reflected in Fig 6. That is to say, for the SAR-SHIP-SET data set, the WA-CNN algorithm can also obtain good ship target detection results, and its performance is also better than that of FCN, U-Net and DeepLabv3+ algorithms.

### C. Analysis of network parameters

**Fig 9:**
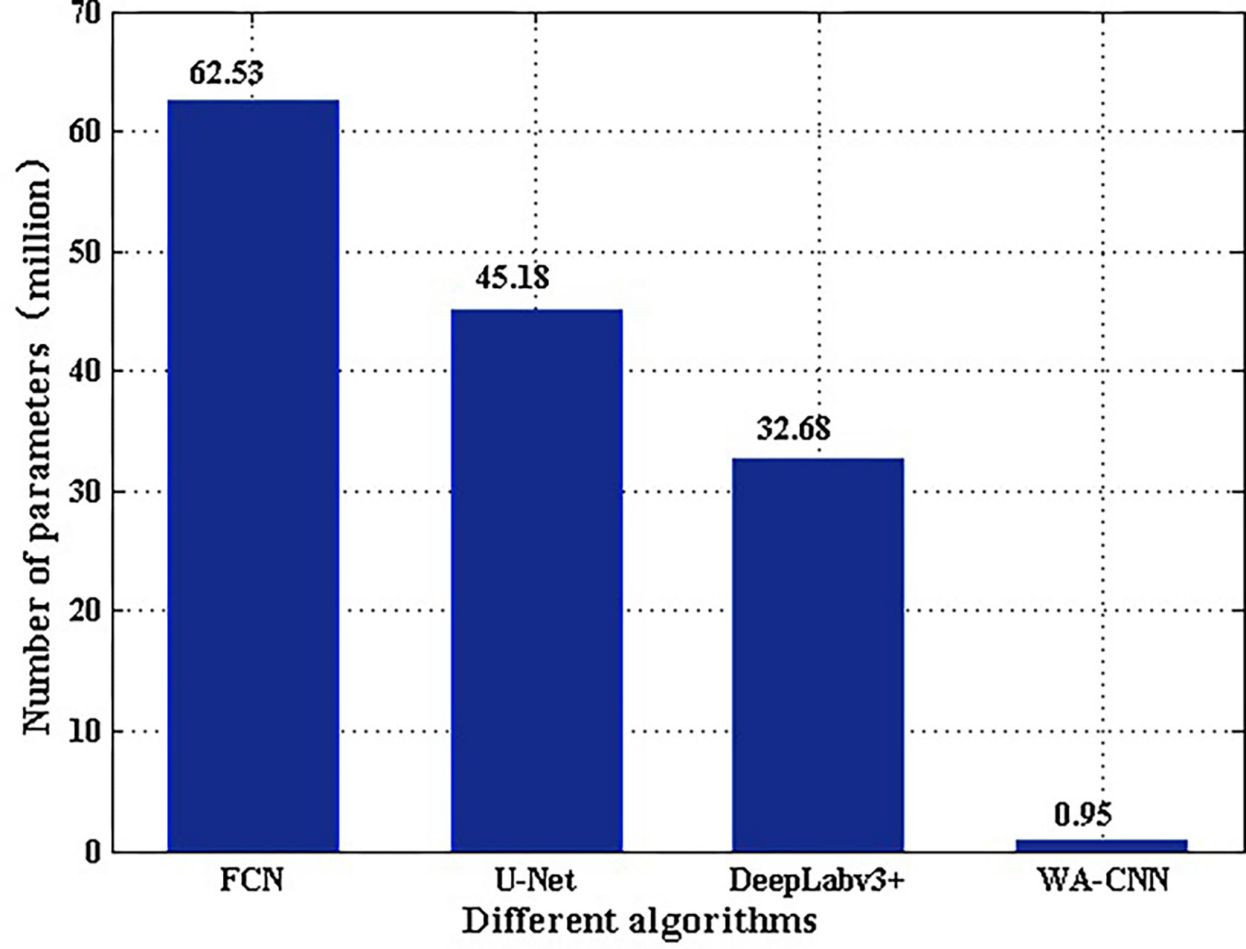
Comparison of parameters of different algorithms.

The complexity of the network is determined by the number of network parameters. The larger the parameter numbers are, the more complex the network is. Network parameters are affected by two factors. One is the depth of the network; another is the structure of the network. The parameters of FCN, U-Net, DeepLabv3+ and WA-CNN are compared and analyzed, and their results are shown in Fig 9. It is very obvious in Fig 9 that the parameters of WA-CNN algorithm are much less than those of other three algorithms.

In terms of network depth, the design of the deep network follows the principle that the length and width of the feature map become half of the original after each down sampling, and the depth of the feature map is doubled. The more down sampling, the deeper the depth of the network and feature maps, which means more convolution kernels and parameters. In the FCN, U-Net, and DeepLabv3+ algorithms, because the depth of the network and feature maps are very deep, the feature maps in the middle layer are simply superimposed, so the utilization of these feature maps with very deep depth is very low. In the WA-CNN algorithm, the deepest depth of the network is only 256. Compared with the previous three algorithms, the depth and the amount of parameters are much reduced, and the network detection performance is enhanced through wavelet pooling and attention mechanism.

In the structure of the WA-CNN algorithm, the network structure consists of an encoder and a decoder. The wavelet pooling layer is embedded in the encoder, and the attention mechanism is introduced in the decoder. The pooling layer only accounts for 5%-8% of the total parameters of the entire network, so the changes in the parameters are mainly determined by the attention mechanism.

The following analyzes the effect of the attention mechanism on the parameters. The attention mechanism replaces a normal convolution. If the position of the attention mechanism is normal convolution, the parameter value is 2*CH*_IN_×3×3×*CH*_OUT_, (*CH*_IN_ = *CH*_OUT_). The attention mechanism consists of three convolution processes. The parameter number of the first convolution process is 2*CH*_IN_×3×3×*CH*_OUT_, and the parameter number of other two convolution processes is *CH*_IN_×3×3×*CH*_IN_/4. It can be clearly found that the parameter amount of the attention mechanism is increased by 25% compared with the normal convolution, but compared with the entire network, the increase in the parameter amount of the attention mechanism in the decoder is negligible.

### D. Impact of network hyper parameters

In Eq (5), the contribution ratio of attention mechanism in the whole network is represented by different values of super parameter *ε* Therefore, changing the contribution ratio of attention mechanism is equivalent to finding the appropriate *ε* value, which can make the detection effect of the whole network reach the best. Next the data of SAR-SHIP-SET is taken as an example to illustrate. In the process of experiment, different detection results can be obtained by setting different *ε* values, that is, embedding appropriate attention mechanism. Since the parameters can be added to the network in the process of forward propagation and backward propagation, it means that the parameters are a learnable variable and can be corrected every iteration. Therefore, the value of the hyper parameters with the best performance can be obtained automatically by iterative training network.

Table 1 shows the evaluation parameter values of the corresponding result image obtained by ship target detection on the SAR image shown in Fig 7(A)–7(J) when different attention mechanisms are injected into the network. The evaluation parameters are still SE, SP, ACC and AUC, and the final value is the mean value after all the images are taken. For example, the value of SE in the second row and second column of Table 1 is 0.7562, which means that when *ε* is equal to 0. The SE value is calculated for the result image after processing all the images in Fig 7, and then the average value is taken. It can be seen that when the value of super parameter *ε* is 0, it indicates that there is no attention mechanism in the network. At this time, the values of SE, SP, ACC and AUC are the minimum. With the increasing degree of attention mechanism, that is, the value of hyper parameter increases, and the value of each evaluation parameter is increasing. It can be seen that when the attention mechanism is added to the network, the detection effect of the network is obviously different. When the value of the super parameter is 0.2, the performance of the network is not improved much. When the value is 0.6, the performance of the network is improved more, and then the attention mechanism is increased, and the performance of the system is improved slowly. For example, when *ε* = 0.6 and *ε* = 1, the values of each evaluation parameter are almost the same, indicating that the network performance has not changed much. This shows that attention mechanism has a great impact on the performance of the network, and the contribution ratio of attention cannot be too small or too large. The contribution ratio of attention should not be too small; otherwise the feature extracted by attention mechanism in convolution layer will be weakened. Of course, its value does not need to be too large, because when it reaches a certain level, the performance hardly changes.

**Table 1:** Evaluation parameter values of the test results under different values of super parameter *ε*.

*ε*	SE	SP	ACC	AUC
0	0.7562	0.9523	0.9425	0.9572
0.2	0.7634	0.9787	0.9543	0.9650
0.6	0.8025	0.9843	0.9754	0.9804
1	0.8012	0.9856	0.9759	0.9812

**Fig 10:**
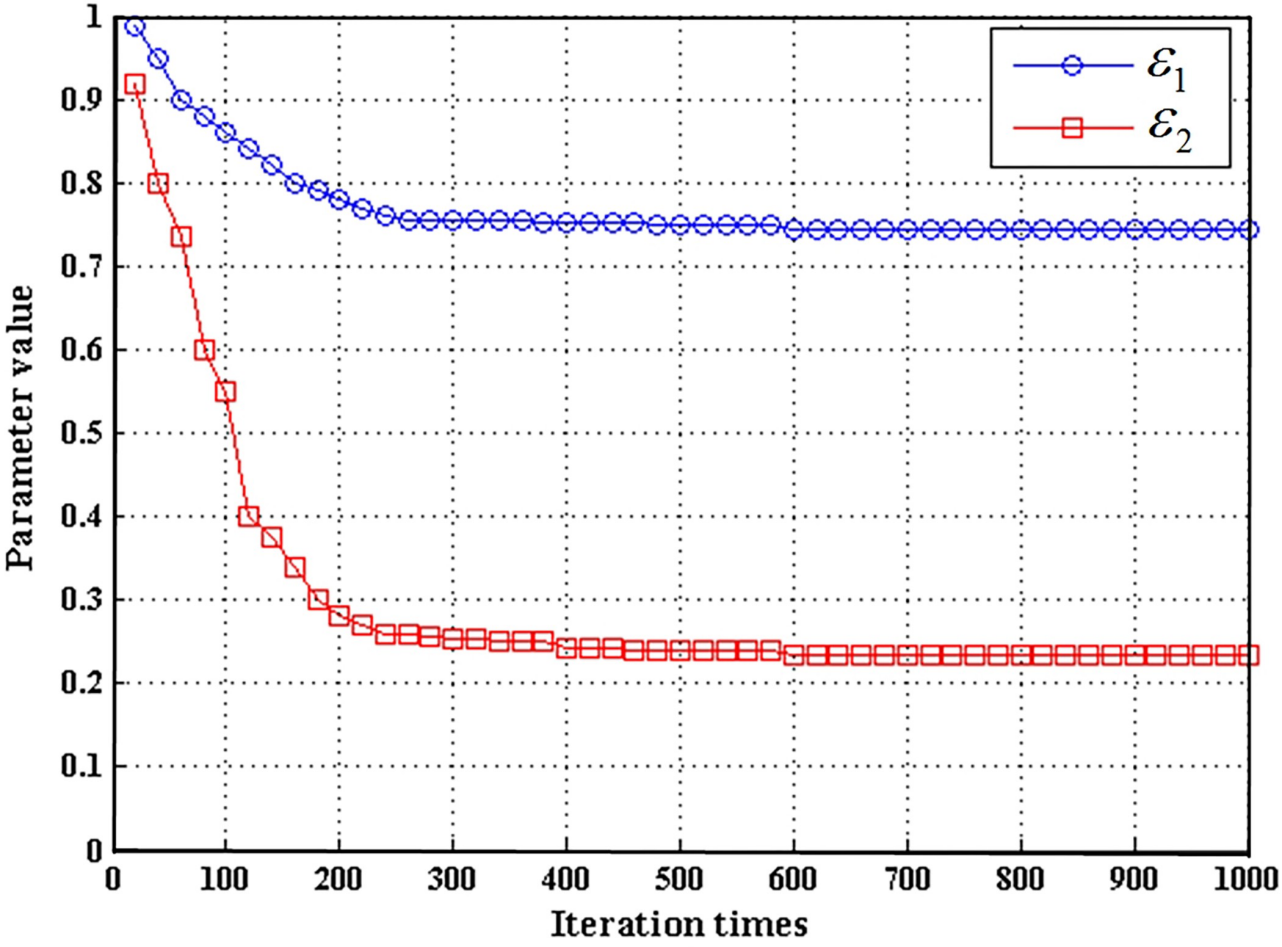
The change curve of super parameter value automatically adjusted with network iteration.

Fig 10 shows the change curve when two super parameters *ε*_1_ and *ε*_2_ are automatically learned and adjusted with the number of network iterations. *ε*_1_ and *ε*_2_ represent the super parameters of the first attention mechanism and the second attention mechanism in the network decoder, respectively. At the beginning of the iteration, the initial value is set to one. When the number of iterations reaches 200, the super parameter value is basically stable. In fact, when the number of iterations reaches 250, the network detection performance is the best, and the values of *ε*_1_ and *ε*_2_ are about 0.75 and 0.25, respectively. This experiment shows that the attention mechanism is effective and the value of super parameter can be determined. At the same time, the super parameter value of the first attention mechanism is larger than that of the second attention mechanism. This is because the first attention mechanism is far from the output of the network and needs to pass through more convolution layers. In order to keep the attention feature from being diluted, it needs a larger super parameter value to make the contribution of attention feature more. On the contrary, the second attention mechanism is close to the output of the network, and only a small attention mechanism is needed to extract better features.

## 5. Conclusions

Due to the interference of various factors during the SAR imaging process, it is difficult to effectively detect and segment ship targets in SAR images. To improve the efficiency of ship target detection, this paper constructed a new WA-CNN method. The basic framework of the WA-CNN network is the U-Net network, but its depth has been significantly reduced. At the same time, the wavelet pooling layer is introduced in the encoder, and the dual-tree complex wavelet transform is used to suppress the speckle noise of SAR image, so that the features can be better maintained than general network structure, which is beneficial to the subsequent effective feature extraction. The attention mechanism layer designed in the decoder is conducive to obtaining global information, making the information more complete, and improving the utilization of the middle layer. The parameter amount of the entire network is very small, only 0.95M. Compared with other similar methods, the complexity is significantly reduced. Many test experiments were carried out through real SAR image datasets, and good experimental results were got, indicating that the WA-CNN proposed in this paper is feasible. In the next step, we will further improve the performance of the network structure and complete the detection of multiple different types of targets.

## Supporting information

S1 Data (DOCX)

S2 Data (DOCX)

S3 Data (DOCX)

S4 Data (DOCX)

S1 Dataset (ZIP)

S2 Dataset (RAR)

S3 Dataset (RAR)

S4 Dataset (RAR)

S5 Dataset (RAR)

S6 Dataset (RAR)

S7 Dataset (RAR)

S1 File (RAR)

## References

[1] Nieto-Hidalgo M, Gallego A J, Gil P, Pertusa A. Two-stage convolutional neural network for ship and spill detection using SLAR images. IEEE Transactions on Geoscience and Remote Sensing, 2018, 56(9): 5217–5230. doi: 10.1109/TGRS.2018.2812619

[2] Snapir B, Waine T W, Biermann L. Maritime vessel classification to monitor fisheries with SAR: demonstration in the north sea. Remote Sensing, 2019, 11(3): 353. doi: 10.3390/rs11030353

[3] Komarov A S, Caya A, Buehner M, Pogson L. Assimilation of SAR ice and open water retrievals in environment and climate change Canada Regional Ice-Ocean Prediction System. IEEE Transactions on Geoscience and Remote Sensing, 2020, 58(6): 4290–4303.

[4] Santos F M, Santos A L C, Violante-Carvalho N, Carvalho L M, Brasil-Correa Y O, Portilla-Yandun J, et al. A simulator of synthetic aperture radar (SAR) image spectra: the applications on oceanswell waves. International Journal of Remote Sensing, 2021, 42(8): 2981–3001.

[5] Dauwalter D C, Fesenmyer K A, Bjork R, Leasure D R, Wenger S J. Satellite and airborne remote sensing applications for freshwater fisheries. Fisheries, 2017, 42(10): 526–537.

[6] Argenti F, Lapini A, Bianchi T, Alparone L. A tutorial on speckle reduction in synthetic aperture radar images. IEEE Geoscience and Remote Sensing Magazine, 2013: 1(3):6–35.

[7] Yoshida T, Ouchi K, Yang C S. Application of MA-ATI SAR for estimating the direction of moving water surface currents in Pi-SAR2 images. IEEE Journal of Selected Topics in Applied Earth Observations and Remote Sensing, 2021, 14:2724–2730.

[8] Gierull C H, Sikaneta I. A compound-plus-noise model for improved vessel detection in non-Gaussian SAR imagery. IEEE Transactions on Geoscience and Remote Sensing, 2017, 56(3): 1444–1453.

[9] Crisp D J. A ship detection system for RADARSAT-2 dual-pol multi-look imagery implemented in the ADSS. 2013 International Conference on Radar, 2013: 318–323.

[10] Torres R, Snoeij P, Geudtner D, Bibby D, Davidson M, Attema E, et al. GMES Sentinel-1 mission. Remote Sensing of Environment, 2012, 120: 9–24.

[11] Pitz W, Miller D. The terrasar-x satellite. IEEE Transactions on Geoscience and Remote Sensing, 2010, 48(2): 615–622.

[12] Marino A, Sanjuanferrer M J, Hajnsek I, Ouchi K. Ship detection with spectral analysis of synthetic aperture radar: a comparison of new and well-known algorithms. Remote Sensing, 2015, 7(5): 5416–5439.

[13] Huang X J, Yang W, Zhang H J, Xia G S. Automatic ship detection in SAR images using multi-scale heterogeneities and an a contrario decision. Remote Sensing, 2015, 7(6): 7695–7711.

[14] Eldhuset K. An automatic ship and ship wake detection system for spaceborne SAR images in coastal regions. IEEE transactions on Geoscience and Remote Sensing, 1996, 34(4): 1010–1019.

[15] Wei S J, Zeng X F, Qu Q Z, Wang M, Su H, Shi J. HRSID: A high-resolution SAR images dataset for ship detection and instance segmentation. IEEE Access, 2020, 8: 120234–120254.

[16] Zhang X T, Chen Z X, Jonathan Wu Q M, Cail L, Lu D, Li X M. Fast semantic segmentation for scene perception. IEEE Transactions on Industrial Informatics, 2018, 15(2): 1183–1192.

[17] Li X, Jiang Y C, Li M L, Yin S. Lightweight attention convolutional neural network for retinal vessel image segmentation. IEEE Transactions on Industrial Informatics, 2020, 17(3): 1958–1967.

[18] Otsu N. A threshold selection method from gray-level histograms. IEEE transactions on systems, man, and cybernetics, 1979, 9(1): 62–66.

[19] Kittler J, Illingworth J. Minimum error thresholding. Pattern recognition, 1986, 19(1): 41–47.

[20] Jin D R, Bai X Z. Distribution information based intuitionistic fuzzy clustering for infrared ship segmentation. IEEE Transactions on Fuzzy Systems, 2019, 28(8): 1557–1571.

[21] Bai X Z, Wang Y F, Liu H N, Guo S. Symmetry information based fuzzy clustering for infrared pedestrian segmentation. IEEE Transactions on Fuzzy Systems, 2018, 26(4): 1946–1959.

[22] Cao X F, Gao S, Chen L C, Wang Y. Ship recognition method combined with image segmentation and deep learning feature extraction in video surveillance. Multimedia Tools and Applications, 2020, 79(13): 9177–9192.

[23] Zhang X Q, Dong G G, Xiong B L, Kuang G Y. Refined segmentation of ship target in SAR images based on GVF snake with elliptical constraint. Remote Sensing Letters, 2017, 8(8): 791–800.

[24] Proia N, Pagé V. Characterization of a Bayesian ship detection method in optical satellite images. IEEE Geoscience and Remote Sensing Letters, 2009, 7(2): 226–230.

[25] Shaik J, Iftekharuddin K M. Detection and tracking of targets in infrared images using Bayesian techniques. Optics & Laser Technology, 2009, 41(6): 832–842.

[26] Long J, Shelhamer E, Darrell T. Fully convolutional networks for semantic segmentation. Proceedings of the IEEE conference on computer vision and pattern recognition, 2015: 3431–3440. 10.1109/TPAMI.2016.2572683 27244717

[27] Nie X, Duan M Y, Ding H X, Hu B L, Wong E K. Attention mask R-CNN for ship detection and segmentation from remote sensing images. IEEE Access, 2020, 8: 9325–9334.

[28] Yekeen S T, Balogun A L, Yusof K B W. A novel deep learning instance segmentation model for automated marine oil spill detection. ISPRS Journal of Photogrammetry and Remote Sensing, 2020, 167: 190–200.

[29] Wang W X, Fu Y T, Dong F, Li F. Semantic segmentation of remote sensing ship image via a convolutional neural networks model. IET Image Processing, 2019, 13(6): 1016–1022.

[30] Chen Y T, Li Y Y, Wang J S, Chen W N, Zhang X Z. Remote sensing image ship detection under complex sea conditions based on deep semantic segmentation. Remote Sensing, 2020, 12(4): 625.

[31] Xiao X W, Zhou Z Q, Wang B, Li L H, Miao L J. Ship detection under complex backgrounds based on accurate rotated anchor boxes from paired semantic segmentation. Remote Sensing, 2019, 11(21): 2506.

[32] Zhang W, He X J, Li W Y, Zhang Z, Luo Y K, Su L, et al. An integrated ship segmentation method based on discriminator and extractor. Image and Vision Computing, 2020, 93: 103824.

[33] Chen L C, Zhu Y K, Papandreou G, Schroff F, Adam H. Encoder-decoder with atrous separable convolution for semantic image segmentation. Proceedings of the European conference on computer vision (ECCV), 2018: 833–851.

[34] Ronneberger O, Fischer P, Brox T. U-net: Convolutional networks for biomedical image segmentation. 2015 International conference on medical image computing and computer-assisted intervention (MICCAI 2015), Lecture Notes in Computer Science, 2015, 12680: 234–241.

[35] Li J W, Qu C W, Sun J. Ship detection in SAR images based on an improved Faster R-CNN. IEEE 2017 SAR in Big Data Era: Models, Methods and Applications (BIGSARDATA), 2017, 1–6.

[36] Wang Y Y, Wang C, Zhang H, Dong Y B, Wei S S. A SAR dataset of ship detection for deep learning under complex backgrounds. Remote sensing, 2019, 11(7): 765.

