# Supplementary material for: SAR ship target detection method based on CNN structure with wavelet and attention mechanism
Source: PLoS One. 2022 Jun 3;17(6):e0265599. doi: 10.1371/journal.pone.0265599 (PMC9165896; doi:10.1371/journal.pone.0265599)
Supplement: S1 Data — (DOCX) [file pone.0265599.s001.docx]

Data of Figure 6

Performance comparison of different algorithms on SSDD data set

| Evaluation parameters | Methods | | | |
| --- | --- | --- | --- | --- |
|  | FCN | U-Net | DeepLabv3+ | WA-CNN |
| SE | 75.53 | 74.72 | 76.46 | 79.21 |
| SP | 92.18 | 92.03 | 93.91 | 97.98 |
| ACC | 92.42 | 92.33 | 94.01 | 96.58 |
| AUC | 92.05 | 92.40 | 94.09 | 96.98 |
