# Supplementary material for: SAR ship target detection method based on CNN structure with wavelet and attention mechanism
Source: PLoS One. 2022 Jun 3;17(6):e0265599. doi: 10.1371/journal.pone.0265599 (PMC9165896; doi:10.1371/journal.pone.0265599)
Supplement: S2 Data — (DOCX) [file pone.0265599.s002.docx]

Data of Figure 8

Performance comparison of different algorithms on SAR-SHIP-SET data set

| Evaluation parameters | Methods | | | |
| --- | --- | --- | --- | --- |
|  | FCN | U-Net | DeepLabv3+ | WA-CNN |
| SE | 76.53 | 76.72 | 77.46 | 80.21 |
| SP | 94.18 | 94.03 | 95.01 | 98.18 |
| ACC | 92.42 | 92.33 | 93.11 | 95.68 |
| AUC | 93.55 | 93.40 | 95.09 | 97.78 |
