# Supplementary material for: SAR ship target detection method based on CNN structure with wavelet and attention mechanism
Source: PLoS One. 2022 Jun 3;17(6):e0265599. doi: 10.1371/journal.pone.0265599 (PMC9165896; doi:10.1371/journal.pone.0265599)
Supplement: S3 Data — (DOCX) [file pone.0265599.s003.docx]

Data of Figure 9

| **Method** | **Parameter numbers (unit: million)** |
| --- | --- |
| FCN | **62.53** |
| U-Net | **45.18** |
| DeepLabv3+ | **32.68** |
| WA-CNN | **0.95** |
