# Supplementary material for: SAR ship target detection method based on CNN structure with wavelet and attention mechanism
Source: PLoS One. 2022 Jun 3;17(6):e0265599. doi: 10.1371/journal.pone.0265599 (PMC9165896; doi:10.1371/journal.pone.0265599)
Supplement: S4 Data — (DOCX) [file pone.0265599.s004.docx]

Data of Figure 10

| Values of $\varepsilon_{1}$ | **[ 0.988, 0.95, 0.90, 0.88, 0.86, 0.84, 0.82, 0.80, 0.79, 0.779,**  **0.768, 0.760, 0.7559, 0.7555, 0.7550, 0.7545, 0.7540, 0.7535, 0.7530, 0.7525,**  **0.7520, 0.7515, 0.7510, 0.7505, 0.7504, 0.7503, 0.7502, 0.7501, 0.7500, 0.7449,**  **0.7448, 0.7447, 0.7446, 0.7445, 0.7444, 0.7443, 0.7442, 0.7441, 0.7440, 0.7440,**  **0.7439, 0.7438, 0.7437, 0.7436, 0.7435, 0.7434, 0.7433, 0.7432, 0.7431, 0.7430]** |
| --- | --- |
| Values of $\varepsilon_{2}$ | **[ 0.918, 0.8, 0.7347, 0.6, 0.55, 0.4, 0.3759, 0.34, 0.30, 0.28,**  **0.27, 0.260, 0.2590, 0.2555, 0.2540, 0.2530, 0.2520, 0.2510, 0.2500, 0.2425,**  **0.2420, 0.2410, 0.2405, 0.2400, 0.2404, 0.2403, 0.2402, 0.2401, 0.2400, 0.2349,**  **0.2348, 0.2347, 0.2346, 0.2345, 0.2344, 0.2343, 0.2342, 0.2341, 0.2340, 0.2340,**  **0.2339, 0.2338, 0.2337, 0.2336, 0.2335, 0.2334, 0.233, 0.2332, 0.2331, 0.2350]** |
| Iteration times | The total number of iterations is 1000 times and the iteration interval for recording values is 20 times. |
